# Effect of Fe_3_O_4_ Nanoparticles on Skin Tumor Cells and Dermal Fibroblasts

**DOI:** 10.1155/2015/530957

**Published:** 2015-05-21

**Authors:** Lirija Alili, Swetlana Chapiro, Gernot U. Marten, Annette M. Schmidt, Klaus Zanger, Peter Brenneisen

**Affiliations:** ^1^Institute of Biochemistry & Molecular Biology I, Medical Faculty, Heinrich Heine University, 40225 Düsseldorf, Germany; ^2^Department of Chemistry, Institute of Physical Chemistry, University of Cologne, 50939 Cologne, Germany; ^3^Institute of Anatomy II, Medical Faculty, Heinrich Heine University, 40225 Düsseldorf, Germany

## Abstract

Iron oxide (Fe_3_O_4_) nanoparticles have been used in many biomedical approaches. The toxicity of Fe_3_O_4_ nanoparticles on mammalian cells was published recently. Though, little is known about the viability of human cells after treatment with Fe_3_O_4_ nanoparticles. Herein, we examined the toxicity, production of reactive oxygen species, and invasive capacity after treatment of human dermal fibroblasts (HDF) and cells of the squamous tumor cell line (SCL-1) with Fe_3_O_4_ nanoparticles. These nanoparticles had an average size of 65 nm. Fe_3_O_4_ nanoparticles induced oxidative stress via generation of reactive oxygen species (ROS) and subsequent initiation of lipid peroxidation. Furthermore, the question was addressed of whether Fe_3_O_4_ nanoparticles affect myofibroblast formation, known to be involved in tumor invasion. Herein, Fe_3_O_4_ nanoparticles prevent the expression alpha-smooth muscle actin and therefore decrease the number of myofibroblastic cells. Moreover, our data show *in vitro* that concentrations of Fe_3_O_4 _ nanoparticles, which are nontoxic for normal cells, partially reveal a ROS-triggered cytotoxic but also a pro-invasive effect on the fraction of squamous cancer cells surviving the treatment with Fe_3_O_4_ nanoparticles. The data herein show that the Fe_3_O_4_ nanoparticles appear not to be adequate for use in therapeutic approaches against cancer cells, in contrast to recently published data with cerium oxide nanoparticles.

## 1. Introduction

Besides an anchorage-independent cell proliferation an important, still treatment-limiting characteristic of malignant tumors is their ability for invasive and metastatic growth [1, 2]. During the invasion process, interactions of tumor cells with the neighbouring interstitial stroma, which is composed of fibroblastic, myofibroblastic, endothelial, and inflammatory cells, as well as extracellular matrix components, play a pivotal role [3, 4]. Molecular mechanisms of tumor-stroma interactions include the secretion of multiple growth factors and cytokines by tumor cells and activated stromal cells which stimulate tumor invasion, tumor development, and neoangiogenesis [5]. Myofibroblasts are modified fibroblasts that express the biomarker alpha-smooth muscle actin (*α*SMA) [6]. The myofibroblastic cell type was originally described in the physiological process of wound healing where it contracts the stroma thereby facilitating wound closure [7, 8]. Meanwhile, it is well known that myofibroblasts are also involved in pathological processes and diseases like fibrosis and cancer [9]. They contribute to tumor progression and are, therefore, often found at the tumor invasion front [10–12]. The interaction between myofibroblasts and cancer cells is dependent on proinvasive growth-promoting factors through paracrine effects [13]. The transition of fibroblasts to myofibroblasts is primarily initiated by transforming growth factor *β*1 (TGF*β*1) [14] and mediated through Smad protein-dependent as well as Smad independent pathways [15]. Previously, we showed that TGF*β*1 induces a reactive oxygen species- (ROS-) mediated pathway leading to formation of myofibroblasts via involvement of protein kinase C (PKC) [16] and NAD(P)H oxidase [17].

Nanoparticles are generally defined as structures with sizes between 1 and 100 nm that have a very large surface-to-volume ratio leading to different, novel properties compared with bulk particles of the same chemical composition [18, 19]. Because of their unique features and the fact that such nanoscale materials are small enough to enter cells and organelles [20, 21], nanoparticles are used for many biomedical approaches* in vitro* and* in vivo* [22]. One example for iron oxide nanoparticle based cancer therapy would be the magnetic fluid hyperthermia therapy (MFH) [23]. Injected magnetic iron oxide nanoparticles are heated by an alternating magnetic field leading to tumor cell death either through apoptosis or necrosis [24, 25]. Although iron oxide nanoparticles are increasingly used for medical purposes, the actual intracellular influence of these structures is not clear till now. As consequence of the increased surface-to-volume ratio, nanoparticles exhibit a potentially higher biological activity compared with larger particles which has been linked to prooxidative but also to antioxidative processes [26–31]. The aim of this study was to determine cell toxicity, myofibroblast development, and tumor invasion, after treatment with Fe_3_O_4_ nanoparticles.

## 2. Materials and Methods

Cell culture media (Dulbecco's modified Eagle's medium (DMEM)) were purchased from Invitrogen (Karlsruhe, Germany) and the defined fetal calf serum (FCS gold) was from PAA Laboratories (Linz, Austria). All chemicals including protease as well as phosphatase inhibitor cocktail 1 and 2 were obtained from Sigma (Taufkirchen, Germany) or Merck Biosciences (Bad Soden, Germany) unless stated otherwise. The protein assay kit (Bio-Rad DC, detergent compatible) was from Bio-Rad Laboratories (München, Germany). Matrigel and polycarbonate cell culture inserts (6.5 mm diameter, 8 *μ*m pore size) were delivered from BD Biosciences (Heidelberg, Germany). The Oxyblot Protein Oxidation Detection Kit was from Millipore (Schwalbach, Germany). The enhanced chemiluminescence system (SuperSignal West Pico/Femto Maximum Sensitivity Substrate) was supplied by Pierce (Bonn, Germany). Monoclonal mouse antibodies raised against human *α*-smooth muscle actin and *α*-tubulin were supplied by Sigma. The following secondary antibodies were used: polyclonal horseradish peroxidase- (HRP-) conjugated rabbit anti-mouse IgG antibody (DAKO, Glostrup, Denmark) and goat anti-rabbit immunoglobulin G antibodies were from Dianova (Hamburg, Germany). Recombinant human TGF*β*1 (rTGF*β*1) was from R&D Systems (Wiesbaden, Germany).

### 2.1. Cell Culture

Human dermal fibroblasts (HDF) were established by outgrowth from foreskin biopsies of healthy human donors with an age of 3–6 years. Cells were used in passages 2–12, corresponding to cumulative population doubling levels of 3–27 [32]. Dermal fibroblasts and the squamous carcinoma cell line SCL-1, originally derived from the face of a 74-year-old woman [33] (generously provided by Professor Dr. Norbert Fusenig, DKFZ Heidelberg, Germany), were cultured as described [34]. Myofibroblasts (MF) were generated by treatment of HDF with recombinant TGF*β*1 (rTGF*β*1) for 48 h in conditioned medium from HDF (CM^HDF^) [16].

### 2.2. Preparation of Conditioned Medium

Conditioned medium was obtained from human dermal fibroblasts (CM^HDF^) and myofibroblasts (CM^MF^). For this, seeded 1.5 × 10^6^ HDF cells were grown to subconfluence (~70% confluence) in 175 cm^2^ culture flasks. The serum-containing medium was removed, and after washing in phosphate-buffered saline (PBS) the cells were incubated in serum-free DMEM or treated with rTGF*β*1 (5 ng/mL) in serum-free DMEM for 48 hours. This medium was removed, and after washing in PBS all cells were incubated in 15 mL serum-free DMEM for further 48 hours before collection of the now called conditioned medium of HDF (CM^HDF^) and myofibroblasts (CM^HDF,TGF*β*1^ = CM^MF^). Conditioned media were used fresh or stored at −20°C for at the most 2 weeks before use [30].

### 2.3. Synthesis and Stabilization of Fe_3_O_4_ Nanoparticles

The synthesis of magnetite nanoparticles on the gram scale was carried out by alkaline precipitation of iron(III) and iron(II) chloride following a method of Cabuil and Massart as described in detail elsewhere [35]. For stabilization, the freshly synthesized nanoparticles were stirred with 420 mL of 2 N nitric acid for 5 min. After washing with distilled water, 90 mL 0.01 N citric acid (CA) was added to the nanoparticles and stirred for 5 min. The particles were magnetically separated from the supernatant and 15 mL of tetramethylammonium hydroxide aqueous solution was added to obtain 3.32 g magnetic nanoparticles Fe_3_O_4_@CA in 92 mL of a stable dispersion at pH 8-9 (yield: 42.5%). The Fe_3_O_4_ content *μ*(Fe_3_O_4_) in dispersion and the magnetic core diameter *d*_c_ were determined via Vibrating Sample Magnetometer (VSM) (*μ*(Fe_3_O_4_) = 2.55 mass%, *d*_c_ = 11.7 nm). DLS: *d*_*h*,*n*_ = 14.3 nm (25°C in H_2_O). Fourier transform infrared spectroscopy (FTIR) (Diamond): *ν* (cm^−1^) = 2357, 2335 (C–N), 1247 (OH), 1098 (C–O), and 1080 (OH) [36].

### 2.4. Surface Modification of Fe_3_O_4_ Nanoparticles

For the immobilization of initiator sites on the particle surface of Fe_3_O_4_@CA, the dispersion was diluted with ethanol to a mass content of 1.0 g·l^−1^, and 1.80 mmol 4-(chloromethyl) phenyltrimethoxysilane (CTS) per gram of Fe_3_O_4_ was added. After stirring for 24 h at ambient temperature, ethanol was removed under reduced pressure at 40°C and the particles were washed with ethanol/acetone (1 : 1) five times. The particles were then redispersed in DMSO, resulting in a Fe_3_O_4_ content *μ*(Fe_3_O_4_) of 6.44 mass % (VSM) in dispersion (yield: 46.4%). The magnetic core diameter *d*_c_ was measured to be 11.1 nm (VSM). The functionalization degree of CPS was determined by EA to be 0.87 mmol CTS on 1.94 g Fe_3_O_4_@CPS. FT-IR (Diamond): *ν* (cm^−1^) = 2357, 2335 (C–N), 1241 (OH), 1115 (Si–O), 1011, and 948 (Si–C) [36].

### 2.5. Surface-Initiated Atom Transfer Radical Polymerization (ATRP) of Functional Polymer Shells

The obtained CPS coated particles served as a macroinitiator for the following ATRP. The synthesis of Fe_3_O_4_@P(O_100_) is described, representatively. Therefore 6 mL of the DMSO-based particle dispersion (0.65 g Fe_3_O_4_@CPS) was mixed with 5 mL of a DMSO solution of 37.3 mg (0.26 mmol) CuBr and 101 mg (0.65 mmol) 2,2-bipyridine (bpy). The polymerization was started by adding 5.83 mmol of the monomer (here: oligo(ethylene glycol) methylether methacrylate (OEGMA)). The mixture was stirred for 24 h at ambient temperature. The obtained viscous magnetic fluid was diluted with 10 mL DMSO to the final ferrofluid. The Fe_3_O_4_ content *μ*(Fe_3_O_4_) in dispersion and the magnetic core diameter *d*_c_ were determined via VSM. The polymer content *χ*_Pol_ in the dried particles was obtained from elemental analysis (EA) and thermogravimetric analysis (TGA) [36].

### 2.6. Particle Transfer to Water/Buffer

The DMSO-based particle dispersion was added dropwise to diethyl ether (Et_2_O). The precipitate was washed five times with Et_2_O/acetone (1 : 1) and was redispersed in distilled water or buffer to obtain an aqueous magnetic fluid [36].

### 2.7. Cell Viability

The cytotoxic effect of Fe_3_O_4_ nanoparticles was measured by MTT (3-(4,5-dimethylthiazol-2-yl)-2,5-diphenyltetrazolium bromide) assay [37]. The activity of mitochondrial dehydrogenases, as indicator of cellular viability, results in formation of a purple formazan dye. Briefly, MTT solution (0.5 mg/mL) was added to the cells treated with different concentrations of Fe_3_O_4_ nanoparticles after washing with PBS. Cells were incubated for an additional 20 min. The medium was removed and the cells were lysed in dimethyl sulfoxide. The formazan formation was measured at 570 nm. The results were presented as percentage of untreated control which was set at 100%.

### 2.8. Cellular Uptake of Nanoparticles

Human dermal fibroblasts (HDF) and squamous cancer cells (SCL-1) in serum-free Dulbecco's Modified Eagle Medium (DMEM) were treated with 350 *μ*M Fe_3_O_4_ nanoparticles for 24 h. Thereafter, cells were harvested and washed with phosphate-buffered saline (PBS) to remove excess media. As the nanoparticles are not detectable by phase contrast microscopy, transmission electron microscopy was used to determine the cellular uptake of Fe_3_O_4_ nanoparticles. For electron microscopy, pelleted samples of Fe_3_O_4_ nanoparticles-treated cells were fixed for 2 h in 4% paraformaldehyde and 2.5% glutaraldehyde (Serva, Heidelberg, Germany) in 0.1 M phosphate buffer at pH 7.4 at room temperature. Next, the pellets were thoroughly washed with four changes of PBS, followed by a postfixation for 60 min in 1% osmium tetroxide (Serva) in PBS. The specimens were dehydrated in a graded series of acetone and embedded in Spurr's medium (Serva) at 70°C for 24 h.

Ultrathin sections were cut from the embedded tissue with a Reichert Ultracut (Vienna, Austria) using a diamond knife. The sections were collected on coated copper grids and subsequently stained with uranyl acetate and lead citrate according to earlier published data [38]. The grids were analyzed using a Hitachi H 600 electron microscope (Düsseldorf, Germany). Documentation was carried out by using an optical system and the Digital Micrograph software (Gatan, Munich, Germany). For light microscopical controls semithin sections were cut and stained with 1% Toluidine blue and 1% Borax [30].

### 2.9. SDS-PAGE and Western Blotting

SDS-PAGE was performed according to the standard protocols published elsewhere [39] with minor modifications. Briefly, cells were lysed after incubation with Fe_3_O_4_ nanoparticles in 1% SDS with 1 : 1000 protease inhibitor cocktail (Sigma; Taufkirchen, Germany). After sonication, the protein concentration was determined by using a modified Lowry method (Bio-Rad DC). 4x SDS-PAGE sample buffer (1.5 M Tris-HCl pH 6.8, 6 mL 20% SDS, 30 mL glycerol, 15 mL *β*-mercaptoethanol, and 1.8 mg bromophenol blue) was added, and after heating, the samples (20–30 *μ*g total protein/lane) were applied to 8% (w/v) SDS-polyacrylamide gels. After electroblotting, immunodetection was carried out (1 : 1000 dilution) of primary antibodies (rabbit monoclonal anti-HIF1*α* and mouse monoclonal anti-*α*-tubulin), 1 : 20000 dilution of anti-mouse/rabbit antibody conjugated to HRP). Antigen-antibody complexes were visualized by an enhanced chemiluminescence system. *α*-tubulin was used as internal control for equal loading.

### 2.10. Invasion Assay

Cell culture inserts (transwells) were overlaid with 125 *μ*g/mL growth factor-reduced matrigel and placed in a 24-well plate. Tumor cells (5 × 10^4^ cells/insert) either untreated or pretreated with Fe_3_O_4_ nanoparticles were seeded on top of the matrigel in serum-free DMEM. Conditioned medium of human dermal fibroblasts (CM^HDF^) or of myofibroblasts (CM^MF^) (see above) was used as chemoattractant in the lower chamber. After 30 h at 37°C, the melanoma cells were rubbed off the upper side of the filter using cotton swabs, and the tumor cells, which invaded to the lower side of the insert, were stained with Coomassie Blue solution (0.05% Coomassie Blue, 20% MeOH, and 7.5% acetic acid). The number of invaded cells was estimated by counting 25 random microscopic fields/insert [16, 30].

### 2.11. Determination of Oxidized (Carbonylated) Proteins: Oxyblot Analysis

Tumor cells were grown to subconfluence on tissue culture dishes. After removal of serum-containing medium, cells were cultured in serum-free medium and either untreated or pretreated for different times with 350 *μ*M Fe_3_O_4_ nanoparticles. As positive control, the cells were treated with 250 *μ*M H_2_O_2_. Thereafter, cells were lysed and carbonyl groups of oxidized proteins were detected with the OxyBlot Protein Oxidation Detection Kit, following the manufacturer's protocol. Briefly, the protein concentration was determined by using a modified Lowry method (Bio-Rad DC). The protein amounts of the samples were aligned. Five *μ*g of this cell lysate was incubated with 2,4-dinitrophenyl (DNP) hydrazine to form the DNP hydrazone derivatives. Labeled proteins were separated by SDS-PAGE and immunostained using rabbit anti-DNP antiserum (1 : 500) and goat anti-rabbit IgG conjugated to horseradish peroxidase (1 : 2000). Blots were developed by enhanced chemiluminescence.

### 2.12. Enzyme-Linked Immunosorbent Assay (ELISA)

By means of the ELISA method, the content of 8-iso prostaglandin F2*α* (8-PGF2a isopropyl, 8-isoprostane) was investigated in cell culture supernatants from SCL-1 cells. The assay was performed using the Acetylcholinesterase Competitive Enzyme Immunoassay kit (Cayman Chemical, Michigan, USA) according to the manufacturer's instructions. This assay is based on the competition between 8-isoprostane and an 8-isoprostane-acetylcholinesterase (AChE) conjugate (8-isoprostane tracer) for a limited number of 8-isoprostane-specific rabbit antiserum binding sites. Because the concentration of the 8-isoprostane tracer is held constant while the concentration of 8-isoprostane varies, the amount of 8-isoprostane tracer that is able to bind to the rabbit antiserum will be inversely proportional to the concentration of 8-isoprostane in the well. This rabbit antiserum-8-isoprostane (either free or tracer) complex binds to the rabbit IgG mouse monoclonal antibody that has been previously attached to the well. The plate is washed to remove any unbound reagents and then Ellman's Reagent (which contains the substrate to AChE) is added to the well. The product of this enzymatic reaction has a distinct yellow color and absorbs strongly at 412 nm. The intensity of this color, determined spectrophotometrically, is proportional to the amount of 8-isoprostane.

### 2.13. Determination of Malondialdehyde (MDA)

MDA is a marker of lipid peroxidation and was determined by HPLC [40] after derivatization with 2-thiobarbituric acid [41]. The HPLC system consisted of a Merck Hitachi L-7100 pump connected with a Merck fluorescence detector (Merck Hitachi; FL Detector L-7480) and a data registration system. Analyses were performed isocratically with a mobile phase composed of 60% phosphate buffer (NaH_2_PO_4_/Na_2_HPO_4_ buffer; 50 mmol/L; pH 6.5) and 40% methanol (v/v) at a flow rate of 1 mL/min and a reversed-phase column (LiChrospher 100 RP18, 5 *μ*m; Merck) protected by a guard column (4.6 × 4.6 mm) of the same stationary phase. Excitation wavelength was 513 nm and emission wavelength 550 nm. MDA levels were calculated by external calibration with 1,1,3,3-tetramethoxypropane, which releases a stoichiometric amount of MDA in an acidic solution. The MDA amount was normalized to the protein content [42].

### 2.14. Statistical Analysis

Means were calculated from at least three independent experiments, and error bars represent standard error of the mean (s.e.m.). Analysis of statistical significance was done by Student's *t*-test or ANOVA with ^*^ *p* < 0.05, ^**^ *p* < 0.01, and ^***^ *p* < 0.001 as levels of significance.

## 3. Results

Herein, the effect of Fe_3_O_4_ nanoparticles in tumor-stroma interaction was studied. We investigated the influence of Fe_3_O_4_ nanoparticles in cultured human dermal fibroblasts and on human squamous carcinoma cells (SCL-1). Fe_3_O_4_ nanoparticles are nontoxic on stromal cells (e.g., fibroblasts) but the cell viability in tumor cells was significantly lowered. Oxidative stress parameters, for example, total reactive oxygen species, carbonylated proteins, and formation of malondialdehyde, were investigated.

### 3.1. Cell Viability

**Figure 1:**
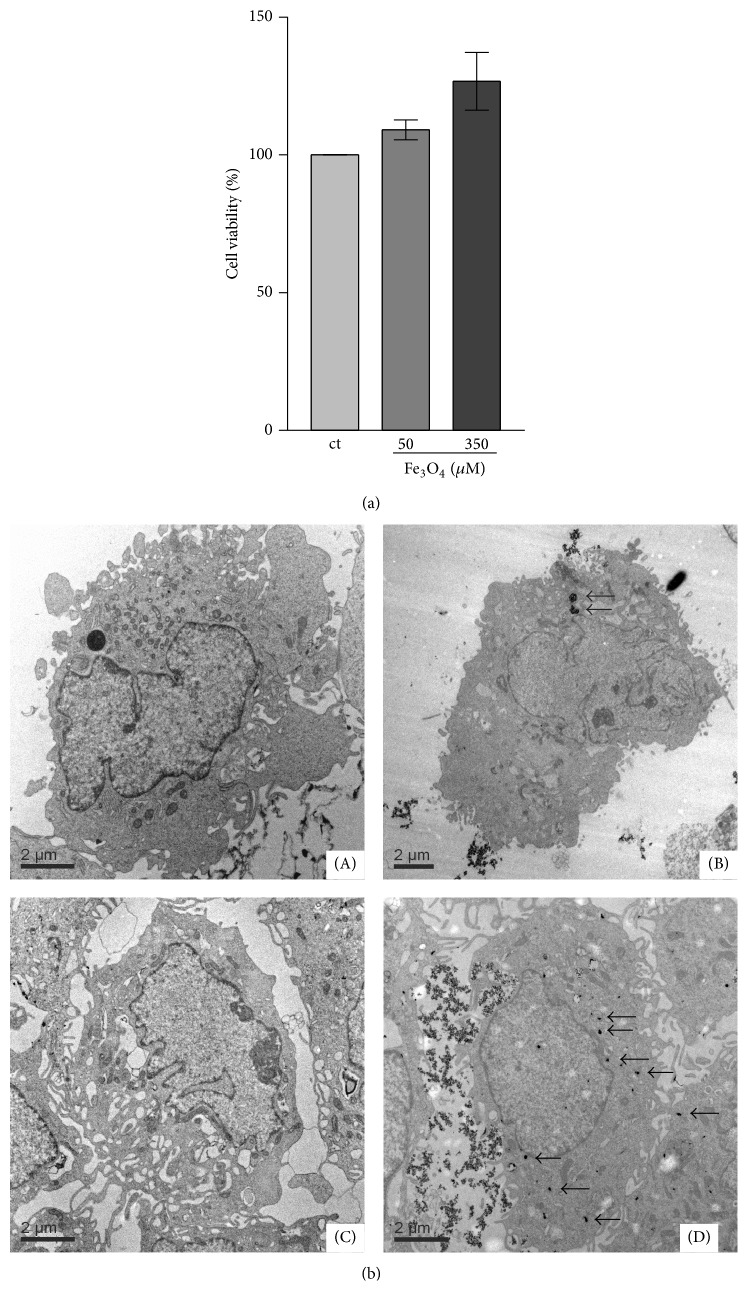
(a) Cell viability of fibroblasts. Cells were incubated with Fe_3_O_4_ nanoparticles. The percentage of living cells was measured after 72 h. *n* = 3. (b) Cellular uptake of Fe_3_O_4_ nanoparticles. Fibroblasts (HDF) (A, B) and SCL-1 tumor cells (C, D) were untreated (A, C) or incubated (B, D) with 350 *μ*M Fe_3_O_4_ nanoparticles for 24 h to determine the cellular uptake of the nanoparticles.

The potential toxicity of Fe_3_O_4_ nanoparticles on human dermal fibroblasts (HDF) was tested. The fibroblasts were incubated with 65 nm-sized polymer-coated Fe_3_O_4_ nanoparticles for 72 h. MTT assays were used to analyze the viability of the cells. Cell viability was evidently not altered after 72 h for these cells (Figure 1(a)).

### 3.2. Cellular Uptake of Fe_3_O_4_ Nanoparticles

The cellular uptake of Fe_3_O_4_ nanoparticles was examined by the use of transmission electron microscopy (TEM).  Figure 1(b) shows the TEM micrographs of human dermal fibroblasts (A, B) and SCL-1 tumor cells (C, D). After treatment of the cells for 24 h with nanoparticles the TEM micrographs of the cells show Fe_3_O_4_ nanoparticles as solid black dots (see arrows) localized in the cytosol (B, D) compared to untreated controls (A, C) (Figure 1(b)). However, the incorporated Fe_3_O_4_ nanoparticles are at least in part agglomerated in the cells.

### 3.3. Effect of Fe_3_O_4_ Nanoparticles on Myofibroblast Development

**Figure 2:**
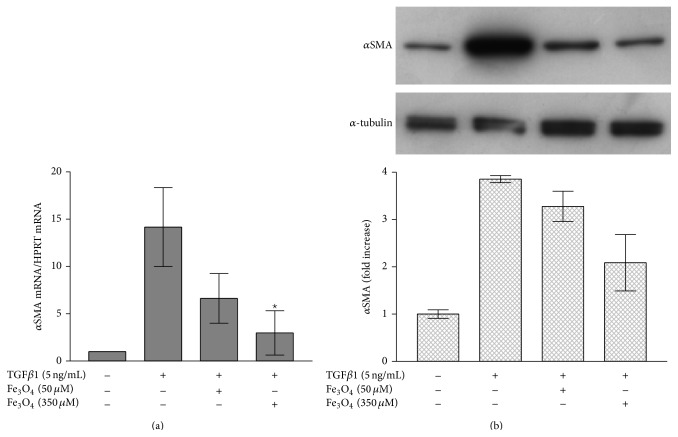
Effect of Fe_3_O_4_ nanoparticles on myofibroblast formation. (a) Gene expression of *α*SMA was analyzed by real-time RT-PCR with normalization against HPRT1. The data represent means ± s.e.m. *n* = 3. CM: conditioned medium. ^*^ *p* < 0.05 versus TGF*β*1 treatment only (Student's *t*-test). (b) Fibroblasts were grown to subconfluence. These cells were either untreated or incubated with 50 *μ*M or 350 *μ*M Fe_3_O_4_ nanoparticles for 24 h before treatment with rTGF*β*1. TGF*β*1 and the Fe_3_O_4_ nanoparticles were present for 48 h. *α*-tubulin served as loading control.  *n* = 3. CM: conditioned medium.

We studied the effect of Fe_3_O_4_ nanoparticles on *α*SMA mRNA level in human dermal fibroblasts using real-time RT-PCR. The “housekeeping” gene HPRT was used as internal control. The *α*SMA mRNA level increased 14 ± 4-fold after 24 h treatment of rTGF*β*1 compared to untreated controls. Pretreatment with Fe_3_O_4_ nanoparticles significantly lowered the rTGF*β*1 mediated transcription of *α*SMA mRNA by 50% (50 *μ*M Fe_3_O_4_) and by 75% (350 *μ*M Fe_3_O_4_) (Figure 2(a)). These data correlated with the *α*SMA protein amount (Figure 2(b)). The *α*SMA protein level was lowered by 50% after preincubation with 350 *μ*M Fe_3_O_4_ nanoparticles (Figure 2(b)).

### 3.4. Fe_3_O_4_ Nanoparticles in Squamous Tumor Cells

**Figure 3:**
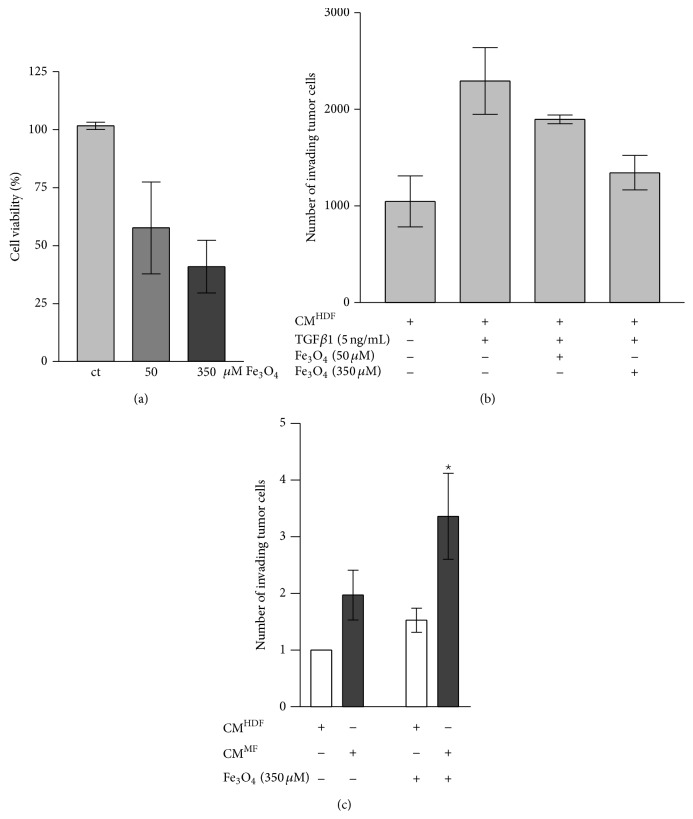
(a) Cell viability of SCL-1 tumor cells. Cells (SCL-1) were incubated with Fe_3_O_4_ nanoparticles. The percentage of living cells after 72 h was measured. *n* = 3. Ct: control (untreated). (b) Involvement of Fe_3_O_4_ nanoparticles in tumor invasion. Conditioned media of HDF (CM^HDF^), myofibroblasts (CM^MF^), and fibroblasts incubated with 50 *μ*M or 350 *μ*M Fe_3_O_4_ nanoparticles and rTGF*β*1 (CM^HDF,TGF,Fe_3_O_4_^) were used to check the invasive capacity of tumor cells. The data represent the mean ± s.e.m. *n* = 3. (c) Direct effect of Fe_3_O_4_ nanoparticles on tumor cells. SCL-1 tumor cells were untreated or incubated with 350 *μ*m Fe_3_O_4_ nanoparticles for 24 h. The invasion assay was performed with conditioned media of HDF (CM^HDF^) and myofibroblasts (CM^MF^). The data represent the mean ± s.e.m. *n* = 3. ^*^ *p* < 0.05 versus CM^MF^ (Student's *t*-test). CM: conditioned medium.

Figure 3(a) displays the result of the MTT assay of SCL-1 cells after incubation with Fe_3_O_4_ nanoparticles for 72 h. 350 *μ*M Fe_3_O_4_ nanoparticles killed tumor cells significantly to 50% but had no toxic effect on the viability of dermal fibroblasts (see Figure 1(a)). These results suggest that Fe_3_O_4_ nanoparticles select between tumor and healthy cells. Therefore it may be used in chemoprevention of squamous tumor cells. The SCL-1 cells which persisted after treatment with 350 *μ*M Fe_3_O_4_, reflect the data of the invasion assays.

### 3.5. Involvement of Fe_3_O_4_ Nanoparticles on Invasive Capacity of Tumor Cells

Myofibroblasts (MF) affect the invasion of tumor cells [16]. Antioxidants inhibit the expression of alpha-smooth muscle actin resulting in prevention of myofibroblast formation [16]. Herein, we checked if Fe_3_O_4_ nanoparticles modulate the invasive capacity of tumor cells. Fe_3_O_4_ nanoparticles treatment inhibits the myofibroblast formation. Conditioned media (CM) of HDF treated with TGF*β*1 and Fe_3_O_4_ nanoparticles (CM^HDF,TGF,Fe_3_O_4_^) were used to check the invasive capacity of tumor cells (Figure 3(b)). The invasion of the squamous tumor cells was 30–50% lowered by using CM^HDF,TGF,Fe_3_O_4_^ compared with CM^HDF,TGF^. Additionally, the direct effect of Fe_3_O_4_ nanoparticles on tumor cells was studied. Therefore, the fibroblasts were not incubated with Fe_3_O_4_ nanoparticles. We used the conditioned medium (CM) of fibroblasts (CM^HDF^) and myofibroblasts (CM^MF^). SCL-1 tumor cells were incubated with 350 *μ*M Fe_3_O_4_ nanoparticles. After 48 h the invasion of the tumor cells was checked. Untreated cells were used as control (Figure 3(c)). Surprisingly, Fe_3_O_4_ nanoparticles significantly increased the invasive capacity by 50% using both CM^HDF^ and CM^MF^. In that context, the direct treatment of SCL-1 tumor cells with cerium oxide nanoparticles showed the opposite effect [30]. In conclusion, even though the prevention of myofibroblast formation by Fe_3_O_4_ nanoparticles resulted in downregulation of tumor invasion, the direct treatment of the SCL-1 cells with the Fe_3_O_4_ nanoparticles increased the invasive capacity.

### 3.6. Reactive Oxygen Species and Carbonylated Proteins

**Figure 4:**
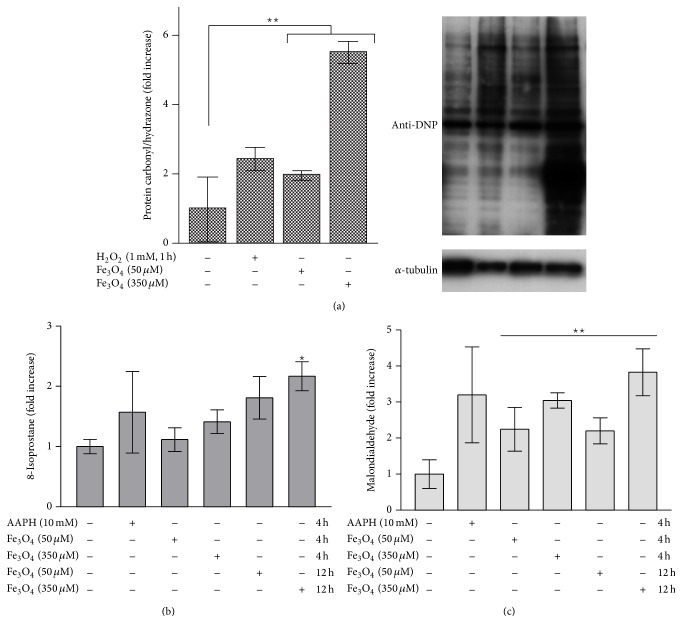
(a) Reactive oxygen species and carbonylated proteins. Tumor cells were untreated or incubated with 350 *μ*M Fe_3_O_4_ nanoparticles for 24 h. Carbonylated proteins were evaluated by western blot analysis. 250 *μ*M H_2_O_2_ was incubated for 1 h and served as positive control and *α*-tubulin served as loading control. *n* = 3. CM: conditioned medium. ^**^ *p* < 0.01 versus untreated control (ANOVA, Dunnett's test). (b) Detection of 8-isoprostane in SCL-1 cell supernatants. Subconfluent SCL-1 cells were incubated with Fe_3_O_4_ nanoparticles (50 *μ*M, 350 *μ*M) for 4 h and 12 h and with 2,2′-azobis(2-amidinopropane) dihydrochloride (AAPH, 10 mM, radical initiator) for 4 h as a positive control. The cell culture supernatants were collected and a competitive ELISA assay applied. The 8-isoprostane concentration of untreated control was set at 1. The data represent the mean ± s.e.m. *n* = 3. ^*^ *p* < 0.05 versus untreated control (CM^SCL^) (Student's *t*-test). (c) MDA formation in SCL-1. MDA formation as a marker for lipid peroxidation was determined by HPLC. Subconfluent SCL-1 cells were treated with Fe_3_O_4_ nanoparticles (50 *μ*M, 350 *μ*M) for 4 h and 12 h and with AAPH (10 mM, radical initiator) for 4 h as a positive control. The data represent the fold increase over control, which was set at 1. The data represent the mean ± s.e.m. *n* = 3. ^**^ *p* < 0.01 versus untreated control (ANOVA, Dunnett's test).

ROS generation plays a great role in cellular viability and invasive capacity of cells. To check whether our results with Fe_3_O_4_ nanoparticles are due to ROS production, we performed a western blot. This special blot detects carbonylated (oxidized) proteins, which is a biomarker for intracellular oxidative stress [43]. SCL-1 tumor cells were incubated with Fe_3_O_4_ nanoparticles for 24 h and the oxyblot was performed. Untreated SCL-1 cells showed a low amount of oxidized proteins. However, H_2_O_2_- and Fe_3_O_4_-treatment significantly increased the amount in tumor cells (Figure 4(a)). The maximum of the level of oxidized proteins was identified after treatment with 350 *μ*M Fe_3_O_4_ nanoparticles with a 5.5-fold increase.

### 3.7. Formation of 8-Isoprostane by Fe_3_O_4_ Nanoparticles

Furthermore, an important parameter for oxidative stress, the lipid peroxidation, was examined [44]. After treatment of the squamous tumor cells with Fe_3_O_4_ nanoparticles, 8-isoprostane, one of several end products of lipid peroxidation, was detected in the cell culture supernatant. The isoprostanes are a family of eicosanoids of nonenzymatic origin produced by the random oxidation of tissue phospholipids by oxygen radicals (8-Isoprostane EIA Kit, Cayman Chemical, Michigan, USA). They are considered as best* in vitro* and* in vivo* markers for oxidative stress and antioxidant deficiency [45, 46]. The content of 8-isoprostane in the supernatant of tumor cells treated with Fe_3_O_4_ compared with untreated control cells is shown in Figure 4(b).

After 4 h of incubation with 50 *μ*M Fe_3_O_4_ nanoparticles, no increase of 8-isoprostane in the culture supernatants of SCL-1 tumor cells was observed compared with untreated control cells, while the treatment with 350 *μ*M Fe_3_O_4_ for 4 h resulted in an increase of the 8-isoprostane level. After 12 h incubation both Fe_3_O_4_ nanoparticle concentrations showed an about 2-fold increase of released 8-isoprostane compared with untreated cells.

### 3.8. Formation of Malondialdehyde by Fe_3_O_4_ Nanoparticles

Lipid peroxidation leads to production of malondialdehyde (MDA). MDA is an unsaturated hydroxyl aldehyde and is generated, like the isoprostanes, as one of several end products during the ROS-induced lipid peroxidation [44]. Treatment of SCL-1 cells with both Fe_3_O_4_ nanoparticle concentrations for 4 h led to a significant increase in the intracellular MDA content compared to untreated control cells (Figure 4(c)). Cells incubated with 50 *μ*M Fe_3_O_4_ showed a 2-fold increase of MDA compared to untreated control cells, while with 350 *μ*M Fe_3_O_4_ a 3-fold increase was detectable in cell lysates. After 12 h, SCL-1 cells treated with 350 *μ*M Fe_3_O_4_ showed an almost 4-fold increased generation of MDA compared to untreated control cells.

Fe_3_O_4_ nanoparticles generate free oxygen radicals and produce significant oxidative stress in squamous tumor cells resulting in a decrease in cell viability, but apparently increase the invasive capacity of cancer cells surviving the treatment with nanoparticles.

## 4. Discussion

An increasing number of different types of nanoparticles are used for applications in the biomedical field, from use as contrast agent to potential carriers for drug delivery. The possible toxic properties of nanoparticles on human health are controversially discussed [18, 47] and further studies are needed to understand and evaluate their function and more specific their toxicity.

Many publications have evaluated the biocompatibility of super paramagnetic iron oxide nanoparticles in different cell types, that is, macrophages [48], endothelial cells [49], and fibroblasts [50, 51]. Experiments on the influence of iron oxide nanoparticles in human dermal fibroblasts, representing stromal cells, and squamous cancer cells are limited.

In comparison with dextran-coated cerium oxide nanoparticles [30], the question was addressed of whether Fe_3_O_4_ nanoparticles have the same bifunctional character like, namely, an antioxidant effect on human dermal fibroblasts and a prooxidative effect in tumor cells.

In this study, we showed that Fe_3_O_4_ nanoparticles prevented TGF*β*1-triggered and ROS-initiated formation of myofibroblasts. The treatment of fibroblasts with the iron nanoparticles is speculated to inhibit the secretion of proinvasive soluble factors and resulted in a significantly lowered invasion of SCL-1 cells. This data correlates with the results obtained with classical antioxidants [16] or redox-active cerium oxide nanoparticles [30, 47]. However, the direct treatment of the tumor cells with the iron nanoparticles increased the invasiveness of a fraction of that cells.

ROS production by Fe_3_O_4_ nanoparticles causes the cytotoxic effect in several cell types [52]. Fe_3_O_4_ is unstable and can easily be oxidized to yield *γ*-Fe_2_O_3_ + Fe^2+^ [53–55]. The free Fe^2+^ ions are able to produce highly reactive hydroxyl radicals (HO^·^, Fenton reaction) by reaction with H_2_O_2_ or O_2_ and Fe^3+^ ions [56] that can modify proteins, lipids, and DNA [52]. Earlier studies described that Fe_3_O_4_ caused an increase in oxidative stress and lipid peroxidation in tumor cells, for example, skin epithelial A431 and lung epithelial A549 [57].

## 5. Conclusion

Fe_3_O_4_ particles with a mean diameter of 65 nm generated reactive oxygen species and, as a consequence, being toxic as well as proinvasive on the fraction of squamous cancer cells surviving the treatment with Fe_3_O_4_ nanoparticles whereas the same concentration does not alter the viability of human dermal fibroblasts which were used as model for stromal cells in skin cancer. These data are in contrast to the recently described effect of cerium oxide nanoparticles on tumor cells [30] indicating that the Fe_3_O_4_ nanoparticles appear not to be adequate for use in therapeutic approaches against cancer cells.
